# Association of ambient carbon monoxide exposure with hospitalization risk for respiratory diseases: A time series study in Ganzhou, China

**DOI:** 10.3389/fpubh.2023.1106336

**Published:** 2023-02-14

**Authors:** Jiahao Song, Weihong Qiu, Xuezan Huang, You Guo, Weihong Chen, Dongming Wang, Xiaokang Zhang

**Affiliations:** ^1^Department of Occupational and Environmental Health, School of Public Health, Tongji Medical College, Huazhong University of Science and Technology, Wuhan, Hubei, China; ^2^Key Laboratory of Environment and Health, Ministry of Education and Ministry of Environmental Protection, and State Key Laboratory of Environmental Health (Incubating), School of Public Health, Tongji Medical College, Huazhong University of Science and Technology, Wuhan, Hubei, China; ^3^First Affiliated Hospital, Gannan Medical University, Ganzhou, China; ^4^Key Laboratory of Prevention and Treatment of Cardiovascular and Cerebrovascular Diseases, Ministry of Education, Gannan Medical University, Ganzhou, China; ^5^School of Public Health and Health Management, Gannan Medical University, Ganzhou, Jiangxi, China

**Keywords:** carbon monoxide, air pollution, hospitalizations, time series study, respiratory diseases, respiratory tract infection (RTI)

## Abstract

**Background:**

Ambient carbon monoxide (CO) exposure is associated with increased mortality and hospitalization risk for total respiratory diseases. However, evidence on the risk of hospitalization for specific respiratory diseases from ambient CO exposure is limited.

**Methods:**

Data on daily hospitalizations for respiratory diseases, air pollutants, and meteorological factors from January 2016 to December 2020 were collected in Ganzhou, China. A generalized additive model with the quasi-Poisson link and lag structures was used to estimate the associations between ambient CO concentration and hospitalizations of total respiratory diseases, asthma, chronic obstructive pulmonary disease (COPD), upper respiratory tract infection (URTI), lower respiratory tract infection (LRTI), and influenza-pneumonia. Possible confounding co-pollutants and effect modification by gender, age, and season were considered.

**Results:**

A total of 72,430 hospitalized cases of respiratory diseases were recorded. Significant positive exposure–response relationships were observed between ambient CO exposure and hospitalization risk from respiratory diseases. For each 1 mg/m^3^ increase in CO concentration (lag0–2), hospitalizations for total respiratory diseases, asthma, COPD, LRTI, and influenza-pneumonia increased by 13.56 (95% CI: 6.76%, 20.79%), 17.74 (95% CI: 1.34%, 36.8%), 12.45 (95% CI: 2.91%, 22.87%), 41.25 (95% CI: 18.19%, 68.81%), and 13.5% (95% CI: 3.41%, 24.56%), respectively. In addition, the associations of ambient CO with hospitalizations for total respiratory diseases and influenza-pneumonia were stronger during the warm season, while women were more susceptible to ambient CO exposure-associated hospitalizations for asthma and LRTI (all *P* < 0.05).

**Conclusion:**

In brief, significant positive exposure–response relationships were found between ambient CO exposure and hospitalization risk for total respiratory diseases, asthma, COPD, LRTI, and influenza-pneumonia. Effect modification by season and gender was found in ambient CO exposure-associated respiratory hospitalizations.

## Introduction

Carbon monoxide (CO), one of the major air pollutants, is an odorless, colorless, tasteless, and non-irritating gas mainly produced by the incomplete combustion of carbon-containing fuels (1). Inhalation through the respiratory tract is the main way ambient CO enters the human body, and circulating CO exerts its toxic effects by binding to heme and altering the function and metabolism of heme protein, which may lead to tissue hypoxia damage and trigger inflammatory and stress responses (2, 3).

The respiratory system is one of the systems most affected by ambient CO exposure. Multiple studies have shown that ambient CO exposure is associated with increased mortality (4) and morbidity (5–7) for total respiratory diseases. A time series analysis evaluating the mortality effects of CO in the Pearl River Delta of China showed that for each 0.5 ppm increase in the 2-day average CO exposure, the excessive risk of respiratory mortality increased by 3.72%. Nevertheless, despite accumulative evidence of adverse effects of ambient CO exposure on total respiratory diseases, the relationship between ambient CO exposure and specific respiratory diseases, such as asthma, chronic obstructive pulmonary disease (COPD), upper respiratory tract infection (URTI), lower respiratory tract infection (LRTI), and influenza-pneumonia, is still lacking or controversial.

For instance, two similar time series studies based on daily hospitalizations for COPD and ambient CO concentration in Hong Kong (8) and Shanghai (9) indicated that short-term ambient CO exposure was associated with reduced risk of COPD hospitalization, suggesting that ambient CO exposure may play an acute protective role against COPD exacerbation. On the other hand, an ecological time series study, including 4,534 COPD hospitalizations in Ahvaz, Iran, found that ambient CO exposure was positively associated with COPD hospitalizations (10). Considering these contradictions, further study is needed to assess the relationship between ambient CO exposure with COPD and other specific respiratory diseases.

Moreover, few analyses have evaluated the health effects of CO exposure through co-pollutant models, and the confounding of other major air pollutants including particulate matter with an aerodynamic diameter of 2.5 μm or less (PM_2.5_), particulate matter with an aerodynamic diameter of 10 μm or less (PM_10_), sulfur dioxide (SO_2_), nitrogen dioxide (NO_2_), and ozone (O_3_), may result in uncertainty in the interpretation of the effect of interest. At the same time, previous studies have shown the potential effect modification by gender, age, and season in the association between ambient CO exposure and respiratory diseases (11, 12), and recognition of effect modification can help to better identify susceptible populations for respiratory diseases from ambient CO exposure.

Consequently, we conducted a time series study in Ganzhou of Jiangxi province in Southeast China to evaluate the association and the exposure–response relationship between ambient CO exposure and the risk of hospitalization for total and specific respiratory diseases including asthma, COPD, upper respiratory tract infection (URTI), lower respiratory tract infection (LRTI), and influenza-pneumonia. Meanwhile, possible confounding co-pollutants and effect modification by age, gender, and season were considered in the current study.

## Methods

### Study area

Ganzhou (24°29′-27°09′N; 113°54′-116°38′E), the largest city in the southern Jiangxi province of China, is characterized by a subtropical monsoon climate. At the time of the study, there were 9.82 million inhabitants over an area of 39,379 square kilometers. The average temperature in 2021 was 19.9°C.

### Hospitalization data

Daily data on hospitalizations from 1 January 2016 to 31 December 2020 were obtained from the medical database of the largest hospital (the First Affiliated Hospital of Gannan Medical University) in Ganzhou. Patient data acquired from the computerized medical record system included age, gender, date of hospitalization, and principal diagnosis on discharge, which was coded using the International Classification of Diseases, 10th Revision. The codes for respiratory diseases were as follows: total respiratory diseases (J00~J98); asthma (J45~J46); COPD (J40~J44); and URTI (J00~J06) including nasopharyngitis, sinusitis, pharyngitis, tonsillitis, laryngitis, tracheitis, and unspecified URTI (13); LRTI (J20~J22) including bronchi, bronchioles, and unspecified LRTI; and influenza-pneumonia (J09~J18).

### Air pollutants and meteorology data

Data on air pollutants in this study were acquired from the National Air Quality Real-time Publishing Platform (https://air.cnemc.cn:18007). The daily average concentrations of ambient PM_2.5_, PM_10_, SO_2_, NO_2_, O_3_, and CO were detected by five fixed monitoring stations in the city. Meteorological data including daily temperature (°C) and relative humidity (%) during the study period were obtained from the China Meteorological Data Service Center (http://data.cma.cn/).

### Statistical analysis

Standard descriptive analysis was conducted to display the distribution of air pollutants, meteorological factors, and daily hospitalizations for respiratory diseases, while the temporal distribution of air pollutants was presented in line charts. Spearman's correlation analysis was used to identify the correlation between air pollutants and meteorological factors.

The generalized additive model was applied to quantify the association between daily ambient CO concentration and daily hospitalizations for respiratory diseases. Quasi-Poisson regression was applied in the model, as daily hospitalizations tended to display an over-dispersed Poisson distribution. A dichotomous variable for public holidays and a categorical variable for the day of the week (DOW) were incorporated into the model to adjust the variation of daily hospitalizations within holidays and each week. Moreover, smoothing terms were used to fit daily hospitalizations in the models to control long-term and seasonal trends of daily hospitalizations and meteorological effects (14). According to previous studies (15–17), we applied 6 degrees of freedom (df) per year for long-term and seasonal trends, 3 *df* each for the same day's temperature (*Tem0*) and relative humidity (*Humid0*). In brief, the model can be represented as follows:


$$\begin{array}{l} Log\left(E\left(Y_{t}\right)\right)=\beta CO+s\left(day,\;df=6/year\times \;no.of\;year\right)\\ \;+s\left(Tem0,\;df=3\right)+s\left(Humid0,df=3\right)+DOW\\ \;+Holiday+\alpha \end{array}$$

where *E*(*Y*_*t*_) represents the estimated daily hospitalizations for respiratory diseases at day *t*. β represents the log-relative risk of hospitalization associated with a 1 mg/m^3^ increase in ambient CO concentration. *s ()* is the restricted smoothing spline function for variables with the non-linear association, *day* indicates the variable of the long-term and seasonal trends, and α is the intercept for the model.

Considering the delayed health effects of air pollutants, we estimated the lag effects of different days in both single-day lag from lag0 to lag7 and multi-day lag from lag0 to lag0–7 (moving average from lag0 to lag7). To improve the comparability of the association between CO exposure and risk of hospitalization for total and specific respiratory diseases, we selected the same CO exposure window for different respiratory diseases in exposure–response relationship analysis, stratified analysis, and sensitivity analysis adjusted for co-pollutants.

Based on the same models that estimated the association between CO exposure and risk of hospitalization for respiratory diseases, the smoothing function with 3 *df* was used to graphically describe the exposure–response relationship between ambient CO concentration and risk of hospitalization for respiratory diseases. Stratified analysis was conducted to assess the potential effect modification by age (minors/adult/elderly), gender (men/women), and season (warm: May–October, cold: November–April) (18, 19). To further quantify the potential effect modification, we calculated the significant differences between subgroups based on the widely used method (20). We also investigated whether the association between CO and hospitalizations was still robust to the adjustment for other co-pollutants including PM_2.5_, PM_10_, SO_2_, NO_2_, and O_3_. Dual- and multi-pollutant models were performed in this study.

In this study, two-sided *P* < 0.05 was considered statistically significant. The generalized additive model was conducted in R 4.0.2 within the “mgcv” package (21). Effect estimates were presented as percentage changes and 95% confidence intervals (CIs) in daily hospitalizations in relation to each 1 mg/m^3^ increase in ambient CO concentration.

## Results

**Figure 1:**
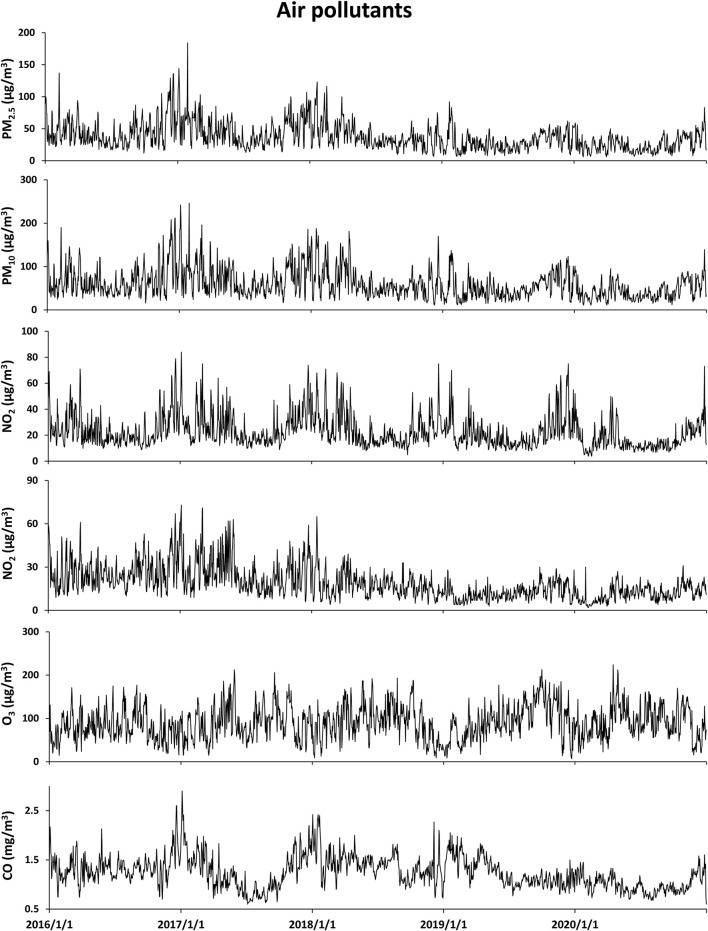
Data for temporal distributions of air pollutants from 1 January 2016 to 31 December 2020 in Ganzhou.

Table 1 summarizes the descriptive statistics on air pollutants, meteorological factors, and hospitalizations for respiratory diseases from 1 January 2016 to 31 December 2020 in Ganzhou. The local average temperature was 20.5°C with an average relative humidity of 75.2%. The concentration of ambient CO ranged from 0.6 to 2.9 mg/m^3^ with an average of 1.2 mg/m^3^, and the temporal distribution of ambient CO and other major air pollutants during the study period is presented in Figure 1. A total of 72,430 hospitalizations for total respiratory diseases were included in this study. The average daily hospitalization numbers were 39.6, 3.1, 13.5, 8.8, 2.6, and 11.8 for total respiratory diseases, asthma, COPD, URTI, LRTI, and influenza-pneumonia, respectively. Daily ambient CO concentration was positively correlated with PM_2.5_ (*r* = 0.44), PM_10_ (*r* = 0.39), NO_2_ (*r* = 0.47), and SO_2_ (*r* = 0.29) while negatively correlated with O_3_ (*r* = −0.15) (Supplementary Table S1).

**Table 1:** Distribution of meteorological factors, air pollutants, and hospitalizations for respiratory diseases from 1 January 2016 to 31 December 2020 in Ganzhou.

**Characteristics**	**Mean ±SD**	**Minimum**	**P25**	**P50**	**P75**	**Maximum**
Temperature (°C)	20.5 ± 8.1	1.0	13.7	21.7	27.8	33.5
Relative humidity (%)	75.2 ± 12	35.5	66.3	75.5	84.5	99.0
PM_2.5_ (μg/m^3^)	37.4 ± 20.8	6.0	23.0	33.0	47.0	184.0
PM_10_ (μg/m^3^)	60.1 ± 33.6	11.0	36.0	52.0	75.0	246.0
NO_2_ (μg/m^3^)	22.8 ± 12.6	4.0	14.0	19.0	28.0	84.0
SO_2_ (μg/m^3^)	18.7 ± 11.2	2.0	11.0	16.0	23.0	73.0
O_3_ (μg/m^3^)	90.5 ± 39.1	7.0	62.0	88.0	116.0	224.0
CO (mg/m^3^)	1.2 ± 0.3	0.6	1.0	1.2	1.4	2.9
**Hospitalizations**						
Total respiratory diseases (*n*/day)	39.6 ± 15.9	5	28	37	49	108
Minors, age ≤ 18, (*n*/day)	13.8 ± 7.0	0	9	13	18	57
Adults, 18 < age ≤ 65, (*n*/day)	12.9 ± 7.4	0	7	11	18	43
Elderly, age > 65, (*n*/day)	13.0 ± 6.8	0	8	12	17	51
Men (*n*/day)	27.1 ± 11.3	3	19	26	34	79
Women (*n*/day)	12.5 ± 6.0	0	8	12	16	38
Asthma (*n*/day)	3.1 ± 2.0	0	2	3	4	13
COPD (*n*/day)	13.5 ± 6.5	0	9	13	18	42
URTI (*n*/day)	8.8 ± 4.1	0	6	8	11	31
LRTI (*n*/day)	2.6 ± 2.1	0	1	2	4	14
Influenza-pneumonia (*n*/day)	11.8 ± 7.9	0	6	9	15	48

SD, standard deviation; P25, P50, and P75, the 25th, 50th, and 75th percentiles; PM_2.5_, particulate matter with aerodynamic diameter < 2.5 μm; PM_10_, particulate matter with aerodynamic diameter < 10 μm; NO_2_, nitrogen dioxide; SO_2_, sulfur dioxide; O_3_, ozone; CO, carbon monoxide; COPD, chronic obstructive pulmonary disease; URTI, upper respiratory tract infection; LRTI, lower respiratory tract infection.

**Figure 2:**
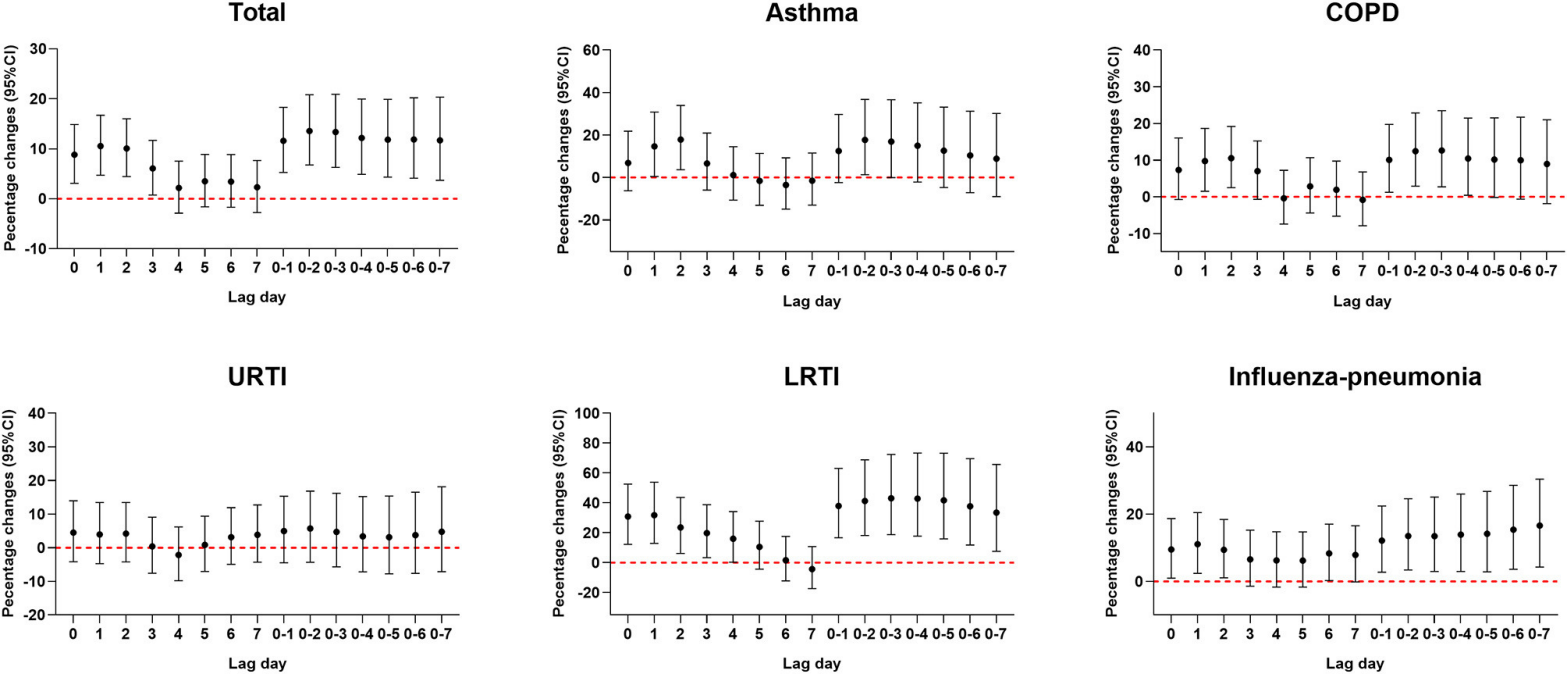
Percent changes (95% CI) in daily hospitalizations for respiratory diseases for each 1 mg/m^3^ increase in carbon monoxide over different lag days. Models were adjusted for long-term and seasonal trends, temperature, relative humidity, public holiday, and DOW (day of the week).

Percentage changes in hospitalizations for respiratory diseases for each 1 mg/m^3^ increase in CO concentration are presented in Figure 2. Significant positive associations of ambient CO concentration with total respiratory diseases, asthma, COPD, LRTI, and influenza-pneumonia were observed in 7-day exposure windows. However, the association of ambient CO with URTI was not found in 7-day exposure windows. Each 1 mg/m^3^ increase in CO concentration at lag0–2 was associated with a 13.56 (95% CI: 6.76%, 20.79%), 17.74 (95% CI: 1.34%, 36.8%), 12.45 (95% CI: 2.91%, 22.87%), 41.25 (95% CI: 18.19%, 68.81%), and 13.50% (95% CI: 3.41%, 24.56%) increment in hospitalizations for total respiratory diseases, asthma, COPD, LRTI, and influenza-pneumonia, respectively.

**Figure 3:**
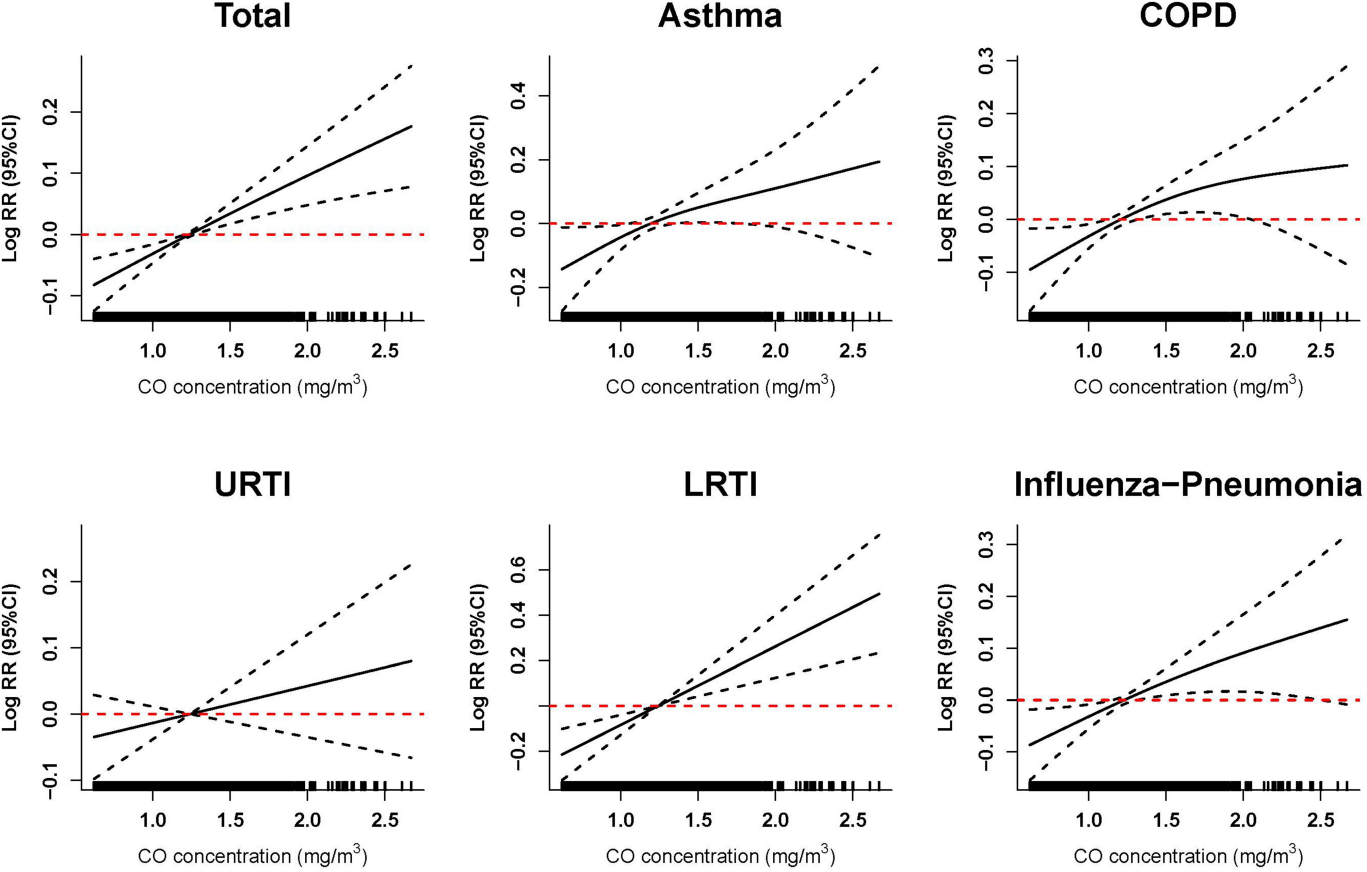
Exposure–response relationship between ambient carbon monoxide and hospitalization risk for respiratory diseases. The vertical scale of the exposure–response curves can be interpreted as the log-relative change from the mean effect of carbon monoxide on the risk of hospitalization. The solid line represents the mean estimate, and the dashed lines represent the 95% confidence intervals. Exposure–response curves were performed at lag0–2 of ambient CO concentration for total and specific respiratory diseases. Models were adjusted for long-term and seasonal trends, temperature, relative humidity, public holiday, and DOW (day of the week).

The exposure–response relationship curves of ambient CO exposure and risk of hospitalization for total and specific respiratory diseases at the highest effect lag days are presented in Figure 3. The curves suggest the positive linear relationship between ambient CO exposure and hospitalization risk for total respiratory diseases, asthma, COPD, LRTI, and influenza-pneumonia. We did not observe any obvious threshold concentration below which ambient CO has no effect or negative effect on the risk of hospitalization for respiratory diseases.

**Figure 4:**
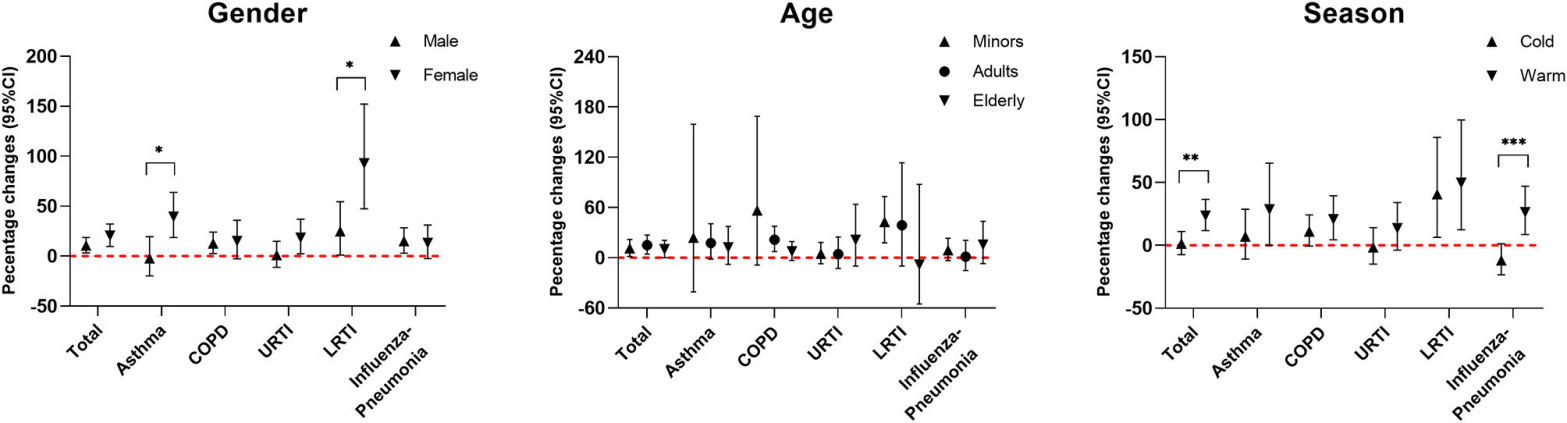
Percent changes (95% CI) in daily hospitalizations for respiratory diseases for each 1 mg/m^3^ increase in carbon monoxide over different subgroups. Stratified analyses were performed at lag0–2 of ambient CO concentration for total and specific respiratory diseases. Models were adjusted for long-term and seasonal trends, temperature, relative humidity, public holiday, and DOW (day of the week). ^*^P < 0.05; ^**^P < 0.01; ^***^P < 0.001.

Figure 4 reveals the percentage change in hospitalizations for total and specific respiratory diseases over different age and gender groups and seasons. Women were more susceptible than men to ambient CO exposure-associated hospitalization risk for respiratory diseases, especially for asthma and LRTI (both *P* < 0.05). Roughly, ambient CO exposure-associated hospitalization risk for respiratory diseases in the cold season was lower than that during the warm season, especially for influenza-pneumonia total respiratory diseases (both *P* < 0.05). However, there was no significant effect modification of age on ambient CO exposure-associated hospitalizations for respiratory diseases.

We also investigated the robustness of the ambient CO exposure effects on hospitalizations for respiratory diseases after adjusting for co-pollutants including PM_2.5_, PM_10_, SO_2_, NO_2_, and O_3_. The results are shown in Table 2. In the multi-pollutant model, we adjusted for PM_10_, SO_2_, NO_2_, and O_3_, while PM_2.5_ was not included due to the strong correlation between PM_2.5_ and PM_10_ [*r* = 0.97]. Generally, the positive association between ambient CO exposure and hospitalizations for asthma, COPD, LRTI, influenza-pneumonia, and total respiratory diseases remained significant after adjustments for PM_2.5_, PM_10_, SO_2_, NO_2_, and O_3_ in the dual-pollutant model and multi-pollutant model. Still, the association of ambient CO exposure with URTI hospitalizations was of no significance after adjustments of co-pollutants.

**Table 2:** Percent changes (95% CI) in daily hospitalizations for respiratory diseases for each 1 mg/m^3^ increase in carbon monoxide with adjustment for co-pollutants.

**Methods**	**Percent changes (95% CI) in daily hospitalizations for respiratory diseases for each 1 mg/m**^**3**^ **increase in CO concentration**					
	**Total**	**Asthma**	**COPD**	**URTI**	**LRTI**	**Influenza-pneumonia**
CO	**13.56 (6.76, 20.79)**	**17.74 (1.34, 36.8)**	**12.45 (2.91, 22.87)**	5.74 (−4.32, 16.85)	**41.25 (18.19, 68.81)**	**13.5 (3.41, 24.56)**
+PM_2.5_	**12.44 (5.4, 19.96)**	**17.72 (3.49, 33.91)**	**12.38 (2.22, 23.55)**	3.62 (−6.85, 15.28)	**35.71 (12.11, 64.27)**	**16.09 (3.78, 29.86)**
+PM_10_	**12.51 (5.43, 20.07)**	**17.71 (3.50, 33.87)**	**12.04 (1.90, 23.17)**	4.40 (−6.20, 16.18)	**33.75 (10.38, 62.06)**	**16.19 (3.85, 30.00)**
+NO_2_	**11.80 (4.90, 19.15)**	**17.80 (3.63, 33.90)**	**11.33 (1.44, 22.19)**	4.31 (−6.01, 15.77)	**37.99 (14.14, 66.82)**	**15.93 (3.65, 29.66)**
+SO_2_	**13.32 (6.36, 20.74)**	**17.83 (3.64, 33.97)**	**13.34 (3.22, 24.44)**	4.15 (−6.14, 15.56)	**38.53 (14.63, 67.42)**	**16.28 (3.95, 30.07)**
+O_3_	**13.63 (6.75, 20.94)**	**17.47 (3.32, 33.56)**	**13.03 (3.04, 23.99)**	5.78 (−4.41, 17.05)	**41.73 (17.57, 70.86)**	**16.59 (4.25, 30.40)**
+PM_10_ + NO_2_ + SO_2_ + O_3_	**12.39 (5.30, 19.95)**	**18.88 (1.29, 39.52)**	**11.87 (1.95, 22.76)**	4.20 (−6.40, 16.01)	**30.84 (8.29, 58.09)**	**12.37 (1.88, 23.94)**

Sensitivity analyses were performed at lag0–2 of ambient CO concentration for total and specific respiratory diseases.

Models were adjusted for long-term and seasonal trend, temperature, relative humidity, public holiday, and DOW (day of the week).

Bold font indicated statistical significance (*P* < 0.05).

## Discussion

In this study, significant positive exposure–response associations of ambient CO exposure with increased hospitalization risk for total respiratory diseases, asthma, COPD, LRTI, and influenza-pneumonia were found. Further sensitivity analysis adjusted for co-pollutants also proved the robustness of these associations. Moreover, the association of ambient CO exposure with hospitalization risk for LRTI among women was stronger, while ambient CO exposure-associated hospitalization risk of total respiratory diseases and influenza-pneumonia in the warm season was more apparent than that in the cold season.

Our identification of the positive associations of ambient CO with hospitalization risk for total respiratory diseases was consistent with much of the previous epidemiological evidence (4–7, 22, 23). A case-crossover study conducted in 26 large cities in China including 916,388 respiratory admissions examined the association between short-term exposure to CO and daily hospitalizations, and results turned out that each 1 mg/m^3^ increase in the CO concentration was associated with a 4.44 (95% CI, 3.97–4.92%) increase in hospitalizations for total respiratory diseases (6). As a review of the mechanism under the carbon monoxide-triggered health effects, CO exposure induced pulmonary edema, immune cell infiltration, and increased COHb, and lung integrity disruption, probably caused by the CO exposure, caused respiratory injury (24). In brief, our study may contribute to identifying the adverse impacts of ambient CO exposure on hospitalizations for respiratory diseases in Ganzhou, China, through the representativeness of the hospital and large sample size.

Nevertheless, the impacts of ambient CO exposure on chronic respiratory diseases such as asthma and COPD remain controversial. Let us take the studies on the association between ambient CO exposure and asthma as an example. The positive association of ambient CO exposure with asthma has been found in the general population, children, or several specific months of the year (25–27) in several studies. On the other hand, several studies indicated that there was no association or even a negative association between ambient CO exposure and asthma (28, 29). Synthesizing the design and methods of the aforementioned studies, these differences may largely be due to the differences in the study population, and children with asthma seemed to be more vulnerable to ambient CO exposure because of their immature lung development. However, the mechanism of the association between ambient CO and asthma was still undetermined. The controversial conclusion on the association of ambient CO and COPD has been stated in the previous part of the article. According to existing mechanism studies, plausible acute protective effects on COPD may be due to the anti-inflammatory action of low-level CO (30). Oxidative stress induced injury of the airway epithelium (31) and inflammation induced pulmonary function reduction (32) were plausible biological mechanisms in the harmful effect of short-term ambient CO exposure on the occurrence or exacerbation of COPD. In this study, positive associations of ambient CO exposure with hospitalizations for asthma and COPD were found through a large sample size and reliable disease classification system, while controlling the ambient CO may reduce the risk of hospitalization for asthma and COPD.

The adverse impact of ambient CO on influenza-pneumonia has been well documented (33, 34). As per the results of a new systematic review and meta-analysis of 21 studies of ambient air pollutants and hospitalizations for pneumonia, each 1 ppm (≈1.2 mg/m^3^) increase in ambient CO concentration was associated with a 4.2 (95% CI: 0.6–7.9%) increase in hospitalizations for pneumonia (35). Another study included 35,862 influenza-like illness (ILI) outpatient visits from January 2015 to November 2017 in Beijing, indicating that CO exposure on lag1 was significantly associated with an increased risk of outpatient visits for ILI (36). There was a viewpoint regarding the mechanisms related to the adverse impact of ambient CO on influenza-pneumonia, that carbon monoxide probably damaged cells by causing lung tissue hypoxia and influencing the energy system in the cells, accordingly increasing the susceptibility to infection (37).

Evidence of ambient CO exposure-associated respiratory infection is still lacking. Tian's research collected daily emergency hospitalization and air pollutant data in Hong Kong, China, from 2001 to 2007 and found that short-term ambient CO exposure was negatively associated with hospitalization risk for respiratory tract infection, suggesting the protective action of low-level ambient CO exposure on the respiratory infection (38). In our study, ambient CO concentration was found to be associated with increased hospitalization risk for LRTI, whereas the association of ambient CO with URTI was non-significant. As far as we knew, we may first come up with the positive association of ambient CO with hospitalizations for LRTI, while the discrepancies from Tian's results may be attributed to different health outcomes and ambient CO levels of 0.72 mg/m^3^ in Hong Kong and 1.2 mg/m^3^ in Ganzhou. The non-significant results on the association of ambient CO exposure and URTI may be because most air pollution-related upper respiratory illnesses are relatively mild and may result in an increase of outpatient visits (39, 40) rather than hospitalizations. Moreover, the diagnosis of URTI in hospitalized patients is mainly due to URTI being associated with other severe systemic diseases.

The exposure–response analysis suggested that hospitalization risk for total respiratory diseases, asthma, COPD, LRTI, and influenza-pneumonia was linearly positively associated with ambient CO exposure. According to existing studies, the positive anti-microbial and anti-inflammatory effects of low ambient CO levels have been found in molecular biology research, and the protective role of low-level ambient CO has also been observed in epidemiological studies (30, 41–43). Meanwhile, the consensus is that high-level ambient CO is harmful to respiratory health. The plausible explanation for the linear positive relationships observed in this study is the relatively higher ambient CO concentration during the study period in Ganzhou. By contrast, the ambient CO concentrations in those studies that reported the protective effects of CO were 0.58 mg/m^3^ in Korea in 2015 (28) and 0.72 mg/m^3^ in Hong Kong from 2001 to 2007 (38), while the average ambient CO concentration was 1.2 mg/m^3^ with a range from 0.6 to 2.9 mg/m^3^ in Ganzhou. Furthermore, different study designs (including different city, hospital, and population structures), social institutions, and cultures may also account for the inconsistencies.

The stratified analysis revealed that ambient CO exposure-associated hospitalization risk for respiratory diseases in the warm season was stronger than that in the cold season, especially for total respiratory diseases and influenza-pneumonia. According to previous studies (44, 45), the stronger associations in warm seasons may be the result of less personal exposure measurement error in relation to more outdoor activity time and natural ventilation (46). Meanwhile, elevated temperatures in warm seasons may cause the thermoregulatory system to activate cardiovascular and respiratory activities to dissipate body heat, and activation directly or indirectly promotes more air pollution entering the body, leading to increased health hazards (11). In addition, women seemed to be more susceptible to ambient CO exposure-associated hospitalization for respiratory diseases. According to previous studies, women showed a more pronounced airway responsiveness, which may be related to increased susceptibility to ambient CO exposure (47). In addition, frequent exposure to cooking fumes may also contribute to women's higher risk of respiratory diseases (48, 49).

There are several strengths of this study. First, our study found the positive associations of ambient CO with total respiratory diseases, asthma, COPD, LRTI, and influenza-pneumonia through a 5-year observation window, adding the epidemiological evidence to elucidate the adverse effects of ambient CO on these specific respiratory diseases. Furthermore, stratified analysis revealed the effect modification by gender and season, providing evidence for identifying susceptible populations and high-risk seasons of ambient CO exposure-associated increased hospitalization risk for respiratory diseases. Nevertheless, there are still some limitations in this study. First, this study was an ecological study based on ambient CO and daily hospitalizations for respiratory diseases, so the assessment of CO exposure was not accurate. All the same, these types of long-term, large sample ecological studies have been widely accepted and have provided epidemiological indications for air pollution exposure-related diseases. Second, as a single-city time series study, the representativeness of our study population was limited, and more multi-center studies are encouraged to be carried out to further confirm our findings on the association of ambient CO with specific respiratory diseases.

## Conclusion

Comprehensively, we found that ambient CO exposure was associated with an increased risk of hospitalization for total respiratory diseases, asthma, COPD, LRTI, and influenza-pneumonia in the linear exposure–response manner. Furthermore, effect modification by season and gender was found in ambient CO exposure-associated hospitalizations for respiratory diseases in this study. These findings may contribute to a better understanding of the health impacts of specific respiratory diseases from ambient CO exposure.

## Data availability statement

The raw data supporting the conclusions of this article will be made available by the authors, without undue reservation.

## Author contributions

JS contributed to the study conception, data collection, statistical analysis, and manuscript drafted. WQ and XH participated in statistical analysis and data collation. YG contributed to the data acquisition. WC contributed to the manuscript drafted and revision. XZ and DW conceived of the study, participated in its design and coordination, and helped to draft the manuscript. All authors reviewed and approved the final manuscript.

## Supplementary material

Supplementary Table S1 Spearman's correlation of air pollutants and meteorological factors. ^*^*P* < 0.05; ***P* < 0.01; ****P* < 0.001.

Supplementary Table S2 Percent changes (95% CI) in daily hospitalizations for respiratory diseases for each 1 mg/m^3^ increase in carbon monoxide over different lag days. Models were adjusted for the long-term trend, temperature, relative humidity, holiday, and DOW (day of the week). The temperature refers to the average temperature on the day of hospitalization and the 2 days preceding it (lag0–2).

Supplementary Table S3 Interaction of ambient CO concentration and meteorological factors on the risk of hospitalization for respiratory diseases. **P* for interaction was estimated by adding a product item between ambient CO concentration and meteorological factor in generalized additive models.

Supplementary Figure S1 Exposure–response relationship between ambient carbon monoxide and hospitalization risks for respiratory diseases for dual and multiple pollution exposures. The vertical scale of the exposure–response curves can be interpreted as the log-relative change from the mean effect of carbon monoxide on the risk of hospitalization. The solid line represents the mean estimate, and the dashed lines represent the 95% confidence intervals. Exposure–response curves were performed at lag0–2 of ambient CO concentration for total and specific respiratory diseases. Models were adjusted for long-term and seasonal trends, temperature, relative humidity, public holiday, and DOW (day of the week).
