# Supplementary material for: Association of ambient carbon monoxide exposure with hospitalization risk for respiratory diseases: A time series study in Ganzhou, China
Source: Front Public Health. 2023 Feb 14;11:1106336. doi: 10.3389/fpubh.2023.1106336 (PMC9972102; doi:10.3389/fpubh.2023.1106336)
Supplement: Supplementary Table S1 — Spearman's correlation of air pollutants and meteorological factors. *P < 0.05; **P < 0.01; ***P < 0.001. [file Data_Sheet_1.docx]

**Supplement materials**

**Table S1.** Spearman’s correlation of air pollutants and meteorological factors.

| Correlation coefficient | Temperature | Relative humidity | PM_2.5_ | PM_10_ | NO_2_ | SO_2_ | O_3_ | CO |
| --- | --- | --- | --- | --- | --- | --- | --- | --- |
| Temperature | 1.00 *** | -0.33 *** | -0.22 *** | -0.14 *** | -0.43 *** | 0.06 | 0.43 *** | -0.32 *** |
| Relative humidity | - | 1.00 *** | -0.21 *** | -0.34 *** | -0.04 *** | -0.29 *** | -0.69 *** | 0.22 *** |
| PM_2.5_ | - | - | 1.00 *** | 0.96 *** | 0.67 *** | 0.69 *** | 0.33 *** | 0.44 *** |
| PM_10_ | - | - | - | 1.00 *** | 0.70 *** | 0.70 *** | 0.43 *** | 0.39 *** |
| NO_2_ | - | - | - | - | 1.00 *** | 0.54 *** | 0.05 *** | 0.47 *** |
| SO_2_ | - | - | - | - | - | 1.00 *** | 0.31 *** | 0.29 *** |
| O_3_ | - | - | - | - | - | - | 1.00 *** | -0.15 *** |
| CO | - | - | - | - | - | - | - | 1.00 *** |

*, *P* < 0.05; **, *P* < 0.01; ***, *P* < 0.001.

**Table S2.** Percent changes (95% CI) in daily hospitalizations for respiratory diseases for each 1mg/m3 increase in carbon monoxide over different lag days.

| Lag days | Percent changes (95% CI) in daily respiratory diseases hospitalizations for each 1mg/m3 increase in CO ^a^ | | | | | |
| --- | --- | --- | --- | --- | --- | --- |
|  | Total | Asthma | COPD | URTI | LRTI | Influenza-Pneumonia |
| Single-day lag |  |  |  |  |  |  |
| lag0 | **12.65 (6.99, 18.62)** | 6.98 (-5.69, 21.34) | 6.09 (-2.3, 15.21) | 1.81 (-7.26, 11.77) | **25.32 (6.5, 47.46)** | **9.46 (0.96, 18.67)** |
| lag1 | **9.18 (3.29, 15.39)** | 11.26 (-2.74, 27.29) | **8.81 (0.5, 17.81)** | 2.88 (-5.91, 12.5) | **26.59 (8.17, 48.14)** | **11.05 (2.38, 20.47)** |
| lag2 | **8.69 (2.99, 14.69)** | **16.12 (1.71, 32.56)** | **10.01 (1.85, 18.81)** | 3.91 (-4.93, 13.56) | **18.93 (1.94, 38.74)** | **9.38 (1.03, 18.42)** |
| lag3 | 3.93 (-1.47, 9.64) | 4.51 (-8.41, 19.25) | 5.73 (-2.09, 14.17) | -2.11 (-10.21, 6.73) | 15.11 (-1.2, 34.12) | 6.55 (-1.45, 15.2) |
| lag4 | -0.64 (-5.7, 4.7) | -1.02 (-12.89, 12.46) | -2.32 (-9.41, 5.33) | -5.25 (-12.91, 3.08) | 12.08 (-3.45, 30.1) | 6.23 (-1.67, 14.76) |
| lag5 | 0.7 (-4.34, 6) | -3.42 (-14.76, 9.44) | 1.15 (-6.05, 8.9) | -1.7 (-9.5, 6.76) | 7.72 (-6.92, 24.67) | 6.2 (-1.67, 14.7) |
| lag6 | 1.73 (-3.36, 7.09) | -5.15 (-16.32, 7.52) | 0.71 (-6.48, 8.45) | 2.09 (-5.99, 10.86) | 1.24 (-12.54, 17.2) | **8.3 (0.24, 17.02)** |
| lag7 | 1.39 (-3.67, 6.72) | -2.85 (-14.25, 10.06) | -1.52 (-8.54, 6.03) | 3.67 (-4.51, 12.57) | -3.16 (-16.34, 12.09) | 7.88 (-0.15, 16.56) |
| Multi-day lag |  |  |  |  |  |  |
| lag0-1 | **9.87 (3.34, 16.82)** | 9.4 (-5.57, 26.73) | 8.79 (-0.31, 18.71) | 2.9 (-6.83, 13.65) | **31.65 (10.64, 56.65)** | **12.15 (2.73, 22.43)** |
| lag0-2 | **11.53 (4.58, 18.94)** | 14.34 (-2.1, 33.54) | **11.1 (1.39, 21.73)** | 3.5 (-6.73, 14.86) | **33.4 (10.97, 60.35)** | **13.5 (3.41, 24.56)** |
| lag0-3 | **10.83 (3.63, 18.53)** | 13.51 (-3.52, 33.54) | **11.11 (1.05, 22.17)** | 2.23 (-8.4, 14.1) | **34.56 (10.95, 63.19)** | **13.47 (2.94, 25.07)** |
| lag0-4 | **9.11 (1.74, 17)** | 11.33 (-5.98, 31.84) | 8.52 (-1.67, 19.76) | -0.23 (-10.91, 11.74) | **34.63 (10.1, 64.61)** | **13.89 (2.95, 26.01)** |
| lag0-5 | **8.25 (0.69, 16.37)** | 8.58 (-8.81, 29.29) | 7.92 (-2.51, 19.47) | -0.94 (-11.9, 11.38) | **33.96 (8.77, 64.99)** | **14.17 (2.84, 26.75)** |
| lag0-6 | **8.04 (0.27, 16.41)** | 5.92 (-11.51, 26.79) | 7.48 (-3.2, 19.34) | -0.31 (-11.67, 12.51) | **30.62 (5.33, 61.99)** | **15.4 (3.57, 28.59)** |
| lag0-7 | 7.98 (0, 16.6) | 4.34 (-13.24, 25.49) | 6.39 (-4.45, 18.46) | 0.87 (-10.93, 14.24) | **27.42 (2.09, 59.03)** | **16.6 (4.26, 30.4)** |

Models were adjusted for long-term trend, temperature, relative humidity, holiday and DOW (day of week). The temperature refers to the average temperature of the day of hospitalization and the two days preceding it (lag0-2).

**Table S3.** Interaction of ambient CO concentration and meteorological factors on the risk of hospitalization for respiratory diseases.

| Meteorological factors | *P* for interaction of ambient CO concentration (lag0-2) with meteorological factors ^*^ | | | | | |
| --- | --- | --- | --- | --- | --- | --- |
|  | Total | Asthma | COPD | URTI | LRTI | Influenza-pneumonia |
| Temperature | **<0.001** | 0.542 | **0.010** | **<0.001** | **0.027** | **0.002** |
| Relative humidity | 0.083 | 0.140 | 0.178 | 0.135 | 0.156 | 0.373 |

^*^ *P* for interaction was estimated by adding a product item between ambient CO concentration and meteorological factor in generalized addictive models.


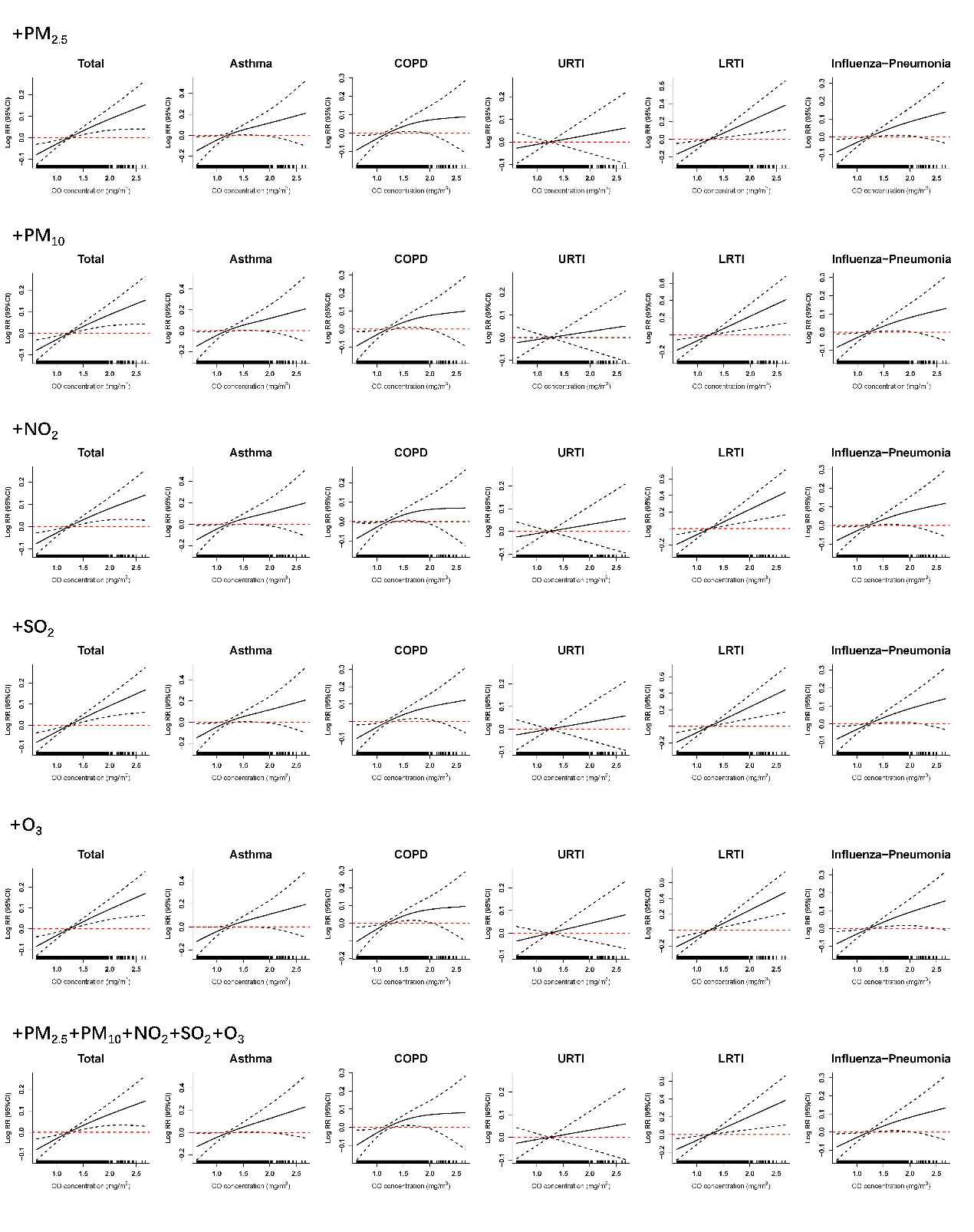


**Figure S1.** Exposure-response relationship between ambient carbon monoxide and hospitalization risks for respiratory diseases for dual and multiple pollution exposures.

The vertical scale of the exposure-response curves can be interpreted as the log-relative change from the mean effect of carbon monoxide on the risk of hospitalization. The solid line represents the mean estimate and the dashed lines represent the 95% confidence intervals.
